# Correlation between the *APOE ε4* genotype, lifestyle habits, and cognitive deficits in Chinese adults over 60: a cross-sectional analysis in China

**DOI:** 10.3389/fpubh.2024.1417499

**Published:** 2024-11-21

**Authors:** Wei Li, XiaoLiang Wang, Lin Sun, Ling Yue, Shifu Xiao

**Affiliations:** ^1^Department of Geriatric Psychiatry, Shanghai Mental Health Center, Shanghai Jiao Tong University School of Medicine, Shanghai, China; ^2^Alzheimer's Disease and Related Disorders Center, Shanghai Jiao Tong University, Shanghai, China; ^3^Division of Psychotic Disorders, Shanghai Mental Health Center, Shanghai Jiao Tong University School of Medicine, Shanghai, China; ^4^Clinical Center for Psychotic Disorders, National Center for Mental Disorders, Shanghai, China

**Keywords:** *APOE ε4*, ways of living, mild cognitive impairment, dementia, community

## Abstract

**Introduction:**

*Apolipoprotein E (APOE) epsilon 4* is regarded as the most significant genetic contributor linked to mild cognitive impairment (MCI) and Alzheimer’s disease (AD). Daily life elements might also influence cognitive abilities to some extent. This research aimed to investigate whether carrying *APOE ε4* alters the effects of lifestyle on cognitive ability.

**Methods:**

The research included 1871 senior community members with *APOE* gene data, all participating in clinical, neuropsychological, and daily living factor assessments. Based on their *APOE ε4* status, they were categorized into two groups: the *APOE ε4* group (*n* = 362) and the *non-APOE ε4* group (*n* = 1,509). Subsequently, a multivariate logistic regression analysis was employed to investigate the link between cognitive deficits and *APOE ε4*, along with lifestyle patterns.

**Results:**

Our research revealed a reduced incidence of MCI (OR = 0.745, 95% CI: 0.587–0.945, *p* = 0.015) and dementia (OR = 0.422, 95% CI: 0.259–0.688, *p* = 0.001) in the non-*APOE ε4* carriers. Furthermore, the general linear regression analysis revealed a notable interplay between *APOE ε4* and sleep disturbances, potentially impacting cognitive deterioration together (*F* = 6.817, *p* = 0.001).

**Conclusions:**

The research indicates that possessing *APOE ε4* alters the impact of everyday life factors on cognitive decline. In addition, there is a significant interaction between *APOE ε4* and sleep disorders, which may jointly lead to the appearance of cognitive impairment.

## 1 Introduction

Despite numerous extensive genome-wide association studies (GWAS) and meta-analyses, the *ε4* allele in the APOE gene continues to be the primary genetic contributor to sporadic Alzheimer’s disease (AD) (1). The presence of *APOE ε4* elevates the likelihood of AD development and concurrently diminishes the onset age of AD (2). Increasingly, data suggests that *APOE ε4* heightens the likelihood of Alzheimer’s disease development through amplified toxic impacts and diminished protective capabilities (3). Beyond Alzheimer’s disease, *APOE ε4* is also linked to mild cognitive impairment (MCI) (4, 5), typical aging (6, 7), and other neurodegenerative conditions like Lewy body dementia (LBD) (8) and vascular dementia (VD) (9). Yet, the precise function of *APOE ε4* in these illnesses remains unclear.

Because dementia is thought to begin decades before clinical symptoms appear, interventions targeting risk factors in non-Alzheimer’s and even middle-aged adults may prevent or delay the onset of cognitive decline (10). Previous studies have explored the link between the Mediterranean diet and Alzheimer’s disease, with diets low in salt, fat, fruits and vegetables thought to be associated with cognitive status (11, 12). In addition to diet, other modifiable lifestyle, such as smoking, depression, hypertension, diabetes mellitus, physical inactivity, low educational attainment, and obesity in middle age, may also play a significant role in the development of dementia (13, 14). Therefore, we propose a comprehensive theory that a “healthy lifestyle” such as a Mediterranean diet, non-smoking, light to moderate alcohol consumption (1–15 g/day for women; 1–30 g/day for men), moderate or vigorous exercise, and active treatment of chronic diseases such as hypertension, diabetes, and depression are associated with cognitive function. An unhealthy lifestyle of high salt, high fat, high sugar diet, sedentary, excessive smoking, excessive alcohol consumption, poor blood sugar and blood pressure control can lead to cognitive decline (15). Similarly, even if all of the above healthy lifestyle patterns or unhealthy lifestyle patterns are not realized, individual or isolation factors may be related to cognitive function. It must be clarified here that the present study was intended only to examine some aspects of the theory, not all.

However, it is not known whether lifestyle influences cognitive function vary by carrying different *APOE* genotypes. In Licher et al.’s (16) study, they demonstrated that favorable modifiable risk profiles were associated with a lower risk of dementia compared to poor genetic risk in individuals with moderate to low genetic risk. In Rosenich et al. (17) study, they found that aging and *APOE ε4* were associated with an increased rate of hippocampal volume (HV) loss and decline in episodic memory (EM). However, the Rodriguez et al. (18) study found that in the general population aged 40–79, the *APOE* gene’s dementia risk variation did not alter the relationship between lifestyle factors and cognitive performance. Large differences between epidemiological studies can be attributed to possible common factors such as differences in sample size, demographic characteristics of participants (including age and sex), method design, and comorbidities (19).

In this study, we used data from the Shanghai Brain Health Cohort Study to investigate whether *APOE* gene changes the impact of lifestyles (e.g., smoking, drinking alcohol, tea drinking, eating habits, characteristic food consumption habits and chronic diseases) on cognitive function. We hypothesized that (1) certain lifestyle patterns (such as smoking, and alcohol consumption) may have an impact on cognitive function; (2) *APOE ε4* genotype may alter the impact of certain lifestyle factors on cognitive function.

## 2 Methods

All participants were from the Shanghai Brain Health Cohort Study, which has been described in detail in our previous studies (20). Simply put, 1,871 community older adults with *APOE* gene data were included in the current study. Of these, 649, or 34.7 percent, were men. Their average age was 69.31 ± 7.87 years, average years of schooling was 10.56 ± 3.64 years, and mean body mass index (BMI) was 23.97 ± 3.37 kg/m^2^. All subjects underwent comprehensive physical examinations, daily habits surveys, clinical data collection, and neuropsychological evaluation. They will also be asked to fill out a standardized questionnaire that includes daily living habits (such as smoking, alcohol drinking, tea drinking, surfing the Internet, etc.), eating habits (such as whether they eat fruit, whether they eat ginger, etc.), and surveys of chronic diseases (such as hypertension, diabetes, depression, sleep duration, etc.).

The research program complies with the Helsinki Declaration ethical guidelines and was approved by the Medical Committee of the Shanghai Mental Health Center. Written informed consent was obtained from all participants.

### 2.1 Clinical assessment

Clinical assessments in all subjects were conducted by two experienced attending physicians. MCI diagnosis was based on Petersen’s criteria: (1) subjective cognitive complaints and were best narrated by family members; (2) neuropsychological tests showing objective cognitive impairment; (3) the ability to maintain daily life; and (4) without dementia (21). Dementia was diagnosed on the basis of Diagnostic and Statistical Manual of Mental Disorders IV (DSM IV) (22, 23): (1) impaired ability to work or perform daily activities; (2) reduced function and performance compared to before; (3) cognitive decline that cannot be explained by delirium or other mental illness; and (4) cognitive or behavioral disorders involving at least two aspects: memory, executive function, visuospatial abilities, speech functions, personality, and behavior changes. The final diagnosis was based on information obtained from semi-structured interviews with the participant’s medical history, standard physical, neurological, and psychiatric examinations.

### 2.2 Neuropsychological tests

#### 2.2.1 Montreal cognitive assessment (MoCA)

Montreal Cognitive Assessment (MoCA) is a screening instrument developed by Kang et al. (24) to detect cognitive impairment. MoCA has a total score of 30, including memory, visuospatial ability, executive ability, language ability, orientation, etc. Based on previous studies, the optimal thresholds for the MoCA comprehensive index of screening for MCI were ≤75 years old, education ≤6 years was 19.5; >75 years old, education ≤6 years was 15.5; and education >6 years was 24.5 (25).

### 2.3 *APOE* genotyping

All participants stopped eating after 9 pm and collected peripheral blood between 7 am and 9 am the following day. Genomic DNA was extracted from blood cells using the Blood Genome DNA Extraction kit (Qiagen NV, Venlo, Netherlands) (after high-speed centrifugation), and *APOE* genotypes were determined by polyploid-amplified-refractory mutation system polymerase chain reaction (PCR). According to method above (26), *APOE ε4* types include ε2/ε4, ε3/ε4, and ε4/ε4, while the class of *non-APOE ε4* types includes ε2/ε2, ε2/ε3, and ε3/ε3.

### 2.4 Lifestyle assessment

Trained interviewers collect data through face-to-face interviews and complete ongoing training before the study begins. Lifestyles we surveyed included smoking, alcohol drinking, tea drinking, eating habits (such as fruit and ginger), surfing the Internet, and information about chronic disease (such as hypertension, diabetes, depression). These variables were selected because previous literature has indicated that all of these factors are strongly associated with cognitive function (27–32). In general, smoking, alcohol consumption, chronic diseases such as hypertension, diabetes, depression, etc., were linked to poorer cognitive function, while tea, vegetables, ginger consumption and surfing the internet were linked to better cognitive performance.

In the current study, smokers were defined as those who smoked more than 100 cigarettes a day in their lifetime, while non-smokers were defined as those who never smoked or smoked fewer than 100 cigarettes in their lifetime (33). A standard unit of alcohol is defined as 14 g of pure alcohol, and five units a day is considered light to moderate alcohol consumption. Non-drinkers are defined as those who never or rarely drink alcohol (for example, holiday drinking, drinking less than one standard unit at a time) (34). Tea drinker was defined whether respondents drank tea more than three times a week. The question is that “over the past month, you have typically had tea several times a week” (35). Since our previous studies have only shown that green tea could help with cognition, in the current study, we are only looking at the effects of green tea on cognitive function (35, 36). Through the Food Frequency Questionnaire (FFQ) (37), we obtained information on participants’ dietary consumption habits and frequency of consumption of fruits and ginger. Investigators would ask, “Do you eat fruit?.” Responses were recorded as daily (people who ate fruit at least once a day), weekly (people who ate fruit 1–6 times a week) or monthly (for those who ate fruit less than once a week). Those who ate fruit at least once a week are considered fruit eaters. The same true of ginger consumption. In the current study, ginger consumption was defined as eating ginger (as a dish) at least once a week, primarily as a food, not a mere flavoring agent. Ginger was listed as a separate variable because previous studies have suggested it may have cognitive protection (38, 39). Surfing the Internet was defined as whether respondents used the Internet and/or e-mail. The question is “Do you use the internet or email?” How often do you surf the Internet?” People who use the internet less than once every 3 months or never go online will be classed as non-internet users (40). Sleep duration surveys are conducted primarily through self-reporting, including: too little sleep (less than 5 h), too much sleep (more than 8 h), and normal sleep (5–8 h). The question is “How many hours of sleep do you get each night.” Diabetes status is determined based on self-reported diagnosis by a physician or treatment with insulin and/or oral hypoglycemic agents. Fasting blood glucose level was ≥7.0 mmol/L (126 mg/dL) or oral glucose tolerance test 2-h value was ≥11.1 mmol/L may also be considered for T2DM (41). Hypertension status was based on self-reported or oral treatment with antihypertensive drugs. Their average systolic blood pressure (SBP) ≥140 mmHg and/or diastolic blood pressure (DBP) ≥90 mmHg was also considered hypertension (42). In addition, depression status was based on self-reporting or oral antidepressant treatment.

## 3 Statistical analysis

The representation of continuous variables was in the form of mean ± standard deviation (SD), while categorical variables were denoted as percentages. Subjects were categorized into groups with *APOE ε4* carriers (*n* = 362) and those without (*n* = 1,509), depending on their *APOE ε4* gene status. Initially, the study employed ANOVA to examine continuous factors like age, education, BMI, and MoCA. Subsequently, the chi-square test evaluated categorical elements including gender, smoking habits, alcohol consumption, tea use, dietary patterns, fruit intake, ginger consumption, surfing the internet, sleep length, hypertension, diabetes, depression, and cognitive categorization between the two groups. The Bonferroni method was employed to account for the effects of multiple tests. Subsequently, we employed multivariate logistic regression to evaluate the link between a solitary lifestyle element and cognitive abilities (MCI and dementia), considering *APOE* status, age, education, BMI, and gender as additional variables. Additionally, to examine if the *APOE ε4* genotype influenced the link between cognitive abilities and lifestyle, multivariate adjusted logistic regression models were employed in subgroup studies. Ultimately, the impact of *APOE ε4* and lifestyle interplay on cognitive abilities was examined using the general linear regression analysis model. Every analysis utilized IBM SPSS Statistics for Windows 22.0, with all *p*-values determined at a two-tailed significance threshold of 0.05.

## 4 Results

### 4.1 Characteristics of the participants

Table 1 displays the features of the research samples. The present study encompassed 1,871 individuals, comprising 362 carriers of *APOE ε4* and 1,509 carriers of *non-APOE ε4*. Within this group, 649 individuals were males, making up 34.7 percent of the overall count. The average age stood at 69.31 ± 7.87 years, with an average educational duration of 10.56 ± 3.64 years, and a mean body mass index of 23.97 ± 3.37 kg/m^2^. People diagnosed with *APOE ε4* showed a reduced tendency to consume ginger, decreased internet usage, a higher probability of experiencing sleep issues, and a greater chance of mild cognitive impairment or dementia (*p* < 0.05).

**Table 1:** Baseline characteristics of the 1,871 participants by *APOE ε4* genotype.

Characteristics	Total sample (n = 1871)	*APOE ε4* genotype		
		Carrier (n = 362)	Non-carrier (n = 1,509)	*p*-value
Age, years	69.31 ± 7.87	69.56 ± 7.77	69.25 ± 7.90	0.505
Education, years	10.56 ± 3.64	10.44 ± 3.64	10.59 ± 3.65	0.459
BMI, kg/m^2^	23.97 ± 3.37	23.93 ± 3.40	23.98 ± 3.37	0.791
Males, *n*(%)	649(34.7)	114(31.5)	535(35.5)	0.158
Smoker, *n*(%)	329(17.6)	59(16.3)	270(17.9)	0.539
Alcohol drinker, *n*(%)	260(13.9)	46(12.7)	214(14.2)	0.499
Tea drinker, *n*(%)	851(45.5)	161(44.5)	690(45.7)	0.681
Eating habit				
Vegetarianism	357(19.1)	78(21.5)	279(18.5)	0.245
Meat-based	71(3.8)	10(2.8)	61(4.0)	
Combination of meat and vegetables	1,443(77.1)	274(75.7)	1,169(77.5)	
Eat fruit	1739(92.9)	344(95.0)	1,395(92.4)	0.087
Eat ginger	488(26.1)	78(21.5)	410(27.2)	0.028*
Surfing the internet	160(8.6)	18(5.0)	142(9.4)	0.006*
Sleep duration problems				
Oversleep	4(0.2)	3(0.8)	1(0.1)	0.010*
Sleep too little	482(25.8)	101(27.9)	381(25.2)	
No abnormalities	1,358(74.0)	258(71.3)	1,127(74.7)	
Hypertension	1,063(56.8)	194(53.6)	869(57.6)	0.174
Diabetes	459(24.5)	77(21.3)	382(25.3)	0.118
Depression	34(1.8)	5(1.4)	29(1.9)	0.661
Cognitive grouping				
Mild cognitive impairment	827(44.2)	175(48.3)	652(43.2)	0.001*
Dementia	84(4.5)	27(7.5)	57(3.8)	
Normal	960(51.3)	160(44.2)	800(53.0)	
MoCA	21.06 ± 5.11	21.15 ± 5.02	20.70 ± 5.46	0.133

*means *p* < 0.05.

### 4.2 Relationship between single lifestyle factors and cognitive impairment

Table 2 displayed the correlation between ginger consumption, surfing the internet, and issues with sleep length with cognitive deficits. Upon adjusting for factors like age, gender, education, *APOE ε4*, and BMI, it was discovered that insufficient sleep significantly correlated with an increased likelihood of MCI (Odds Ratio = 1.275, 95% confidence interval: 1.106–1.600, *p*-value = 0.036). Likewise, our findings indicate a significant correlation between insufficient sleep and an increased likelihood of developing dementia (OR = 1.880, 95% confidence interval: 1.094–3.232, *p* = 0.022). Nonetheless, the revised model revealed no notable links between ginger consumption or surfing the internet, and the presence of MCI or dementia.

**Table 2:** Association between single lifestyle factor and cognitive impairment.

Independent variable	Logistic regression, OR of MCI (95%CI)			
	Unadjusted model	*p*	Adjusted model	*p*
Eat ginger	0.872(0.706–1.077)	0.204	0.924(0.740–1.155)	0.489
Surfing the Internet	0.764(0.548–1.064)	0.111	0.747(0.524–1.065)	0.117
Sleep too little	1.405(1.134–1.740)	0.002*	1.275(1.016–1.600)	0.036*

Independent variable	Logistic regression, OR of dementia (95%CI)			
	Unadjusted model	*p*	Adjusted model	*p*
Eat ginger	0.475(0.259–0.873)	0.016*	0.604(0.306–1.192)	0.146
Surfing the Internet	0.110(0.015–0.797)	0.029*	0.209(0.030–1.459)	0.114
Sleep too little	2.006(1.248–3.223)	0.004*	1.880(1.094–3.232)	0.022*

Controlled for age, sex, education, *APOE ε4*, BMI. *means *p* < 0.05.

### 4.3 Association between *APOE ε4* genotypes and lifestyles and cognitive impairment

The multivariable logistic regression analysis, treating diagnosis as the dependent factor and *APOE ε4* classification as the independent, revealed that individuals without *APOE ε4* had a reduced likelihood of developing MCI (OR = 0.745, 95% confidence interval: 0.587–0.945, *p* = 0.015) and dementia (OR = 0.422, 95% confidence interval: 0.259–0.688, *p* = 0.001). Our examination of subgroups with the *APOE ε4* gene revealed that educational levels (OR = 0.871, 95% confidence interval: 0.810–0.938, *p* < 0.001), BMI (OR = 0.913, 95% confidence interval: 0.847–0.985, *p* = 0.019), and tea consumption (OR = 0.548, 95% confidence interval: 0.339–0.886, *p* = 0.014) correlated with a reduced likelihood of MCI; age (OR = 1.145, 95% confidence interval: 1.061–1.236, *p* = 0.001) was linked to an increased risk of dementia, whereas hypertension (OR = 0.157, 95% confidence interval: 0.047–0.527, *p* = 0.003) correlated with a decreased risk of dementia.

Our examination of subgroups lacking the *APOE ε4* gene revealed a correlation between age (OR = 1.016, 95% confidence interval: 1.001–1.032, *p* = 0.031) and an increased likelihood of MCI, whereas education (OR = 0.093, 95% confidence interval: 0.874–0.933, *p* < 0.001) correlated with a reduced probability of MCI. Factors such as age (Odds Ratio = 0.004, 95% Confidence Interval: 1.017–1.093, *p*-value = 0.004), insufficient sleep (Odds Ratio = 2.612, 95% Confidence Interval: 1.508–4.522, *p*-value = 0.001), and depression (Odds Ratio = 7.410, 95% Confidence Interval: 2.706–20.291, *p*-value<0.001) correlated with an increased likelihood of dementia. Conversely, education (Odds Ratio = 0.887, 95% Confidence Interval: 0.828–0.950, *p*-value = 0.001), tea consumption (Odds Ratio = 0.506, 95% Confidence Interval: 0.277–0.925, *p*-value = 0.027), and fruits consumption (Odds Ratio = 0.309, 95% Confidence Interval: 0.143–0.666, *p*-value = 0.003) were linked to a reduced risk of dementia. The findings are displayed in Tables 3, 4.

**Table 3:** Association between multiple lifestyle factors and cognitive impairment in participants with the *APOE ε4* gene.

Variable	*B*	S.E.	Wald	df	*p*	OR	95%CI
MCI							
Education	−0.138	0.037	13.579	1	<0.001*	0.871	0.810–0.938
BMI	−0.091	0.039	5.466	1	0.019*	0.913	0.847–0.985
Age	−0.016	0.018	0.770	1	0.380	0.984	0.949–1.020
Tea drinker	−0.601	0.245	6.017	1	0.014*	0.548	0.339–0.886
Eat fruit	0.161	0.673	0.057	1	0.811	1.175	0.314–4.396
Eat ginger	0.176	0.313	0.317	1	0.573	1.193	0.646–2.205
Surfing the Internet	−0.104	0.461	0.501	1	0.821	0.901	0.365–2.225
Sleep too little	0.178	0.287	0.382	1	0.536	1.195	0.680–2.099
Hypertension	0.153	0.285	0.289	1	0.591	1.166	0.667–2.038
Depression	0.207	0.285	0.527	1	0.468	1.230	0.704–2.149
Dementia							
Age	0.135	0.039	12.070	1	0.001*	1.145	1.061–1.236
Hypertension	−1.850	0.617	8.985	1	0.003*	0.157	0.047–0.527
Education	−0.222	0.093	5.744	1	0.051	0.801	0.668–0.960
BMI	0.035	0.113	0.906	1	0.756	1.036	0.830–1.291
Tea drinker	0.697	0.743	0.879	1	0.348	2.007	0.468–8.609
Eat fruit	–						
Eat ginger	–						
Surfing the Internet	−0.180	1.245	0.021	1	0.885	0.835	0.073–9.584
Sleep too little	1.015	0.727	1.952	1	0.162	2.760	0.664–11.471
Hypertension	1.455	1.369	1.130	1	0.288	4.285	0.293–62.684
Depression	−1.621	0.777	4.353	1	0.370	0.198	0.043–0.906

*means *p* < 0.05.

**Table 4:** Association between multiple lifestyle factors and cognitive impairment in participants without the *APOE ε4* gene.

Variable	*B*	S.E.	Wald	df	*p*	OR	95%CI
MCI							
Age	0.016	0.008	4.652	1	0.031*	1.016	1.001–1.032
Education	−0.102	0.017	37.809	1	<0.001*	0.903	0.874–0.933
BMI	0.010	0.016	0.401	1	0.527	1.010	0.978–1.044
Tea drinker	−0.325	0.128	6.428	1	0.110	0.722	0.562–0.929
Sleep too little	0.211	0.140	2.286	1	0.131	1.235	0.939–1.623
Eat fruit	−0.363	0.234	2.399	1	0.121	0.696	0.440–1.101
Eat ginger	−0.133	0.138	0.927	1	0.336	0.876	0.669–1.147
Surfing the internet	−0.115	0.233	0.246	1	0.620	0.891	0.565–1.406
Hypertension	0.030	0.133	0.050	1	0.823	1.030	0.793–1.338
Depression	−0.250	0.433	0.334	1	0.564	0.779	0.334–1.819
Dementia							
Age	0.053	0.018	8.194	1	0.004*	1.054	1.017–1.093
Education	−0.120	0.035	11.839	1	0.001*	0.887	0.828–0.950
BMI	−0.010	0.047	0.042	1	0.837	0.990	0.903–1.086
Tea drinker	−0.682	0.308	4.899	1	0.027*	0.506	0.277–0.925
Eat fruit	−1.175	0.392	8.992	1	0.003*	0.309	0.143–0.666
Eat ginger	0.378	0.358	1.114	1	0.291	1.460	0.723–2.946
Sleep too little	0.960	0.280	11.745	1	0.001*	2.612	1.508–4.522
Hypertension	−0.023	0.383	0.004	1	0.952	0.977	0.462–2.068
Depression	2.003	0.514	15.183	1	<0.001*	7.410	2.706–20.291
Surfing the Internet	0.932	0.955	0.953	1	0.329	2.540	0.391–16.493

Ultimately, a general linear regression model was employed to explore how the interaction of *APOE ε4* genotype with sleep duration issues impacts general cognitive abilities. This general linear regression model was executed with insufficient sleep as the sole lifestyle factor, as it was the only one significantly linked to both MCI and dementia in the logistic regression studies. Within this framework, the aggregate MoCA score serves as the dependent variable, whereas issues related to sleep duration and the *APOE ε4* genotype are categorized as a constant element. Ultimately, the findings revealed a notable interplay between the *APOE ε4* genotype and issues with sleep duration, potentially impacting cognitive deterioration (*F* = 6.817, *p* = 0.001), and the existence of *APOE ε4* coupled with “insufficient sleep” indicated a potential reduction in MoCA scores (notably, *APOE ε4* by itself had no impact on MoCA scores). Due to the lack of significant positive outcomes (*p* > 0.05) for other factors like ginger consumption and surfing the internet in the general linear regression model, the aforementioned results were omitted.

## 5 Discussion

In the current study, we investigated the relationship between *APOE ε4* genotype, lifestyle and cognitive decline. Unlike other studies, we added some new variables, such as eating habits, eating ginger, sleep duration problems, and internet use, etc. The reason for adding the above variables is that our previous studies have suggested that these variables are associated with cognitive function (43–46). At the same time, ethnic differences are an important factor to consider. Compared with the older adult in Europe and America, the older adult in China may also have different lifestyles (such as drinking tea, eating ginger, and lunch break), leading to differences in dementia incidence and influencing factors. The incidence of dementia, for example, varies by race, as high as 8.5 percent Latin America and 5.14 percent China (47). A meta-analysis found that the *APOE ε4* and *ε2* affect blacks differently form other races (48). Asians have the highest burden of physical and memory impairments and are more likely to show impaired language fluency due to low education (49). In addition, other studies have shown that birth can lead to differences between ethnic groups in Asia, and that social factors may have a different impact on the development of dementia (50). Therefore, through the epidemiological investigation of the older adult in China, we can find new influencing factors of Alzheimer’s disease and provide new theoretical basis for the prevention and treatment of Alzheimer’s disease. Through multiple logistics regression analysis, we finally found that (1) the risk of mild cognitive impairment and dementia was significantly reduced in people who did not carry the *APOE ε4* allele; (2) *APOE ε4* carrying status may affect the impact of everyday life on cognitive decline; and (3) there was a significant interaction between *APOE ε4* and sleep duration problems, both of which may contribute to cognitive impairment.

*The apolipoprotein (APOE) epsilon 4 allele* is the most common genetic risk factor for Alzheimer’s disease (AD), accounting for about 4% of Alzheimer’s disease variation (51). The relationship between *APOE ε4* genotype and cognitive impairment is well studied. For example, a meta-analysis showed that *APOE ε4* was strongly associated with susceptibility to vascular dementia in Chinese population (52). Another meta-analysis showed that *APOE ε2+* was neither a risk factor nor a prevention factor for Parkinson’s disease dementia (PDD), while *APOE ε4+* was a risk factor for PDD (53). In addition, many studies have also shown that *APOE ε4* may adversely affect MCI (54–56). Therefore, our conclusions are consistent. There are several theories about the effects of the *APOE ε4* allele on the brain. One theory is that different *APOE* variants have different effects on the accumulation of amyloid-beta in the brain (57). Another theory is that different *APOE* alleles differ in binding and clearance of Aβ (58). In addition, the study further suggests that e4 variants increase blood–brain barrier damage (59).

Our results showed that the carrying status of *APOE ε4* could change the influence of daily lifestyle on cognitive decline, and the influencing factors of MCI and dementia were significantly different with *APOE ε4* gene carrying status. For example, in our current study, we found that education, BMI and tea drinker were associated with lower MCI odds among participants with the *APOE ε4* gene, while age was associated with higher MCI odds, and education was associated with lower MCI odds among *non-APOE ε4* carriers. However, we found only a significant interaction between *APOE ε4* and sleep duration problems on cognitive impairment, and not no interaction between *APOE ε4* and other variables, such as tea drinking, Internet use, diabetes, hypertension, depression, etc.

Aging is characterized by changes in sleep quality and structure. When sleep changes become apparent, they can produce or accelerate cognitive decline, even in the absence of significant pathological factors (60). Aging changes sleep duration and quality, which is also common in Alzheimer’s disease. The increased in Aβ production and decrease in reduced Aβ clearance were due to the close interplay of Aβ, sleep disturbance and wakefulness. In addition to Aβ, sleep deprivation found in AD may be associated with the effects of tau pathology (61). There is strong evidence linking sleep quality to the onset and development of dementia (62, 63). For example, a cross-sectional study conducted in China showed that poor suboptimal sleep duration and quality in non-institutionalized adults aged 45 years and older (*n* = 10,768) were associated with poor cognitive ability. A state-of-the-art review shows a four-pronged link between sleep disruption and quality of life of people with dementia: physical, social/behavioral, emotional health, and cognition (64). In addition, a systematic study suggested that up to 90 percent of people with Lewy body dementia (LBD) have at least one sleep disorder, such as subjectively poor sleep quality, excessive daytime sleepiness (EDS), and rapid eye movement behavior disorder (RBD) (65).

Interestingly, sleep quality appears to be intrinsically linked to *APOE* genetic polymorphism. For example, a pilot study showed that healthy older adults with a risk allele (*APOE ε4+*) have more sleep complaints or objective evidence of sleep disruption than healthy older adults without a risk allele (*APOE ε4−*) (66). An integrative review have shown that *APOE ε4* was associated with poor sleep quality in terms of sleep efficiency, sleep latency, rapid eye movement, wake after sleep onset, 24-h total sleep time, and the deterioration of nighttime total sleep time in people with mild cognitive impairment (MCI) or AD (67). In addition, a population-based study of older adults in rural China showed a link between sleep problems and dementia and AD, mainly in people with *APOE ε4* (68). Therefore, our conclusions are consistent.

We have to admit that our study has some limitations. First, it is only a cross-sectional study and cannot establish a causal link between *APOE ε4*, a variable of daily life, and cognitive decline; Second, long-term follow-up of these populations is needed to observe the long-term effects of *APOE ε4* and daily life variables on cognitive function; Third, we were not able to uncover the mechanism by which *APOE ε4* interacts with sleep duration problems to influence cognitive decline; Finally, this cross-sectional study includes people with MCI and dementia, and the exposure variable (participation in lifestyles) is collected using self-reported information. Thus, recall bias is present and can interfere with the findings.

In summary, we found that the carrying state of *APOE ε4* can change the influence of daily life variables on cognitive impairment, and there may be a significant interaction between *APOE ε4* and sleep disorders, which jointly promote the occurrence of cognitive impairment. Although many previous studies have pointed out that daily lifestyle may have a certain impact on cognitive function, they have not paid special attention to the regulatory role of *APOE4* on the above effects. Therefore, in this sense, our study has made further extension and expansion to reveal the possible mechanism of *APOE ε4’s* regulation of cognitive function.

## Data Availability

The original contributions presented in the study are included in the article/supplementary material, further inquiries can be directed to the corresponding authors.
